# State-of-the-Art Room Temperature Operable Zero-Bias Schottky Diode-Based Terahertz Detector Up to 5.56 THz

**DOI:** 10.3390/s23073469

**Published:** 2023-03-26

**Authors:** Rahul Yadav, Florian Ludwig, Fahd Rushd Faridi, J. Michael Klopf, Hartmut G. Roskos, Sascha Preu, Andreas Penirschke

**Affiliations:** 1 High Frequency Technology, Mittelhessen University of Applied Sciences, 61169 Friedberg, Germany; 2 Terahertz Devices and Systems, TU Darmstadt, 64283 Darmstadt, Germany; 3 Institute of Physics, Johann Wolfgang Goethe University, 60438 Frankfurt am Main, Germany; 4 Institute of Radiation Physics, Helmholtz-Zentrum Dresden-Rossendorf, 01328 Dresden, Germany

**Keywords:** free-electron laser (FEL), broadband detectors, THz sources, THz radiation detectors, zero-bias Schottky diode, room temperature detectors

## Abstract

We present the characterization of a Zero-bias Schottky diode-based Terahertz (THz) detector up to 5.56 THz. The detector was operated with both a table-top system until 1.2 THz and at a Free-Electron Laser (FEL) facility at singular frequencies from 1.9 to 5.56 THz. We used two measurement techniques in order to discriminate the sub-ns-scale (via a 20 GHz oscilloscope) and the ms-scale (using the lock-in technique) responsivity. While the lock-in measurements basically contain all rectification effects, the sub-ns-scale detection with the oscilloscope is not sensitive to slow bolometric effects caused by changes of the IV characteristic due to temperature. The noise equivalent power (NEP) is 10 pW/$\sqrt{\mathrm{Hz}}$ in the frequency range from 0.2 to 0.6 THz and 17 pW/$\sqrt{\mathrm{Hz}}$ at 1.2 THz and increases to 0.9 μW/$\sqrt{\mathrm{Hz}}$ at 5.56 THz, which is at the state of the art for room temperature zero-bias Schottky diode-based THz detectors with non-resonant antennas. The voltage and current responsivity of ∼500 kV/W and ∼100 mA/W, respectively, is demonstrated over a frequency range of 0.2 to 1.2 THz with the table-top system.

## 1. Introduction

The electromagnetic spectrum from 0.1 to 10 THz was commonly known as THz gap because there were very efficient microwave devices at longer wavelengths and very efficient optical sources and detectors at shorter wavelengths. The lack of powerful sources, sensitive detectors and other measurement equipment had limited the possibility for research in the THz domain despite important applications in the fields of spectroscopy, imaging, communication and security [1,2,3,4]. Since the late 20th century, scientists and researchers have worked rigorously towards the development of more powerful sources, low-noise detectors and measurement systems to bridge this gap [5,6]. An important application of Terahertz detectors is the diagnostics at THz-generating particle accelerator facilities (Free-Electron Lasers, synchrotrons) [7,8,9,10]. Diagnostic techniques on the sub-ps-scale use, e.g., electro-optical sampling (EOS) and optical-rectification (OR), which is a coherent detection mechanism that usually requires a phase-locking of the source and receiver [11]. The ultrafast polarization of electro-optic crystals on the time scale of the THz field enabled the discovery of coherent interactions of the THz fields with matter [12]. The down-side of coherent detection techniques is that they can only be used for measuring individual pulses or they require a rigorous phase-locking of the naturally independent accelerator pulse and the optical signal driving the EO crystal. Direct (incoherent) detectors, in contrast, rectify an incident THz signal and do not require any phase-locking. Golay cells, bolometers and photo-thermal electric (pyroelectric) detectors are examples of thermal THz detectors [13,14,15,16]. These are frequently used to monitor the pulse energy or spatially track the beam emerging from a THz source. Most thermal detection concepts are quite slow with detection constants in the ms-range or longer, as they are operated in or close to thermal equilibrium. Exceptions are, e.g., hot electron bolometers, where the electron gas is not in thermal equilibrium with the lattice. Examples of fast detectors that allow, in principle, sub-ns temporal resolution, are rectifying high electron mobility transistors (HEMTs) [17,18,19] and Schottky diodes [20,21,22,23]. In terms of speed, they are predominantly limited by their RC time constants, which can be as high as 1 THz or even more.

High power and coherent THz radiation generated at accelerator facilities such as Free-Electron Lasers (e.g., FELBE, FELIX, FLASH-THz or the planned UKXFEL) [24,25,26] are frequently used to probe matter in the non-linear regime or in pump–probe experiments. Prior to such experiments, it is important to characterize the THz beam coming from the beam line and to align it to the actual setup [27,28,29,30]. Furthermore, measuring the coherent THz radiation from relativistic electrons can be a powerful tool for determining the temporal characteristics of the bunches in particle accelerators [31,32]. For this purpose, we develop HEMT-based direct THz detectors the and zero-bias Schottky diode [19,20] for beam diagnosis at accelerator facilities [18,19,20], where the latter one is presented in this paper. Due to their compact size and room temperature operation, they are easy to handle and cost-effective.

This paper is divided into five sections. Section 1 introduces THz technology and gives an overview of room temperature THz detectors for various applications, with a focus on THz radiation from Free-Electron Lasers. In Section 2, the Schottky diode-based THz detector developed for this study is discussed, followed by Section 3, in which the experimental setups used are described. Section 4 is dedicated to results and discussion. The conclusions of this paper are explained in Section 5.

## 2. Zero-Bias Schottky Diode THz Detector

The zero-bias Schottky diode (SD) [33,34] used in this project was produced by ACST GmbH [21,22,23]. The Schottky diode is integrated with a log-spiral antenna on a so-called “film-substrate”. The film substrate is a very thin foil (values are classified by ACST GmbH) which is attached to a silicon chip of the size 5×1.6×0.5 mm, having an absolute dielectric constant of $|\varepsilon_{r}|\;=\;11.7$. This helps in reducing parasitic effects, which ultimately plays a crucial role for the detector performance at higher frequencies. Since both the log-spiral antenna and the SD are on a commercial chip provided by ACST GmbH, the specific dimensions of the antenna used in the chip are confidential but the design frequency range is known. The antenna is close to a self-complementary antenna with a radiation resistance of about $R_{A}\;=\;72$ Ω on a GaAs-air interface [35]. Above 2 THz, the antenna dimensions are too large compared to the wavelength; thus, it stops working efficiently [36]. Thus, at higher THz frequencies, the THz wave directly couples to the central electrodes of the detector.

**Figure 1:**
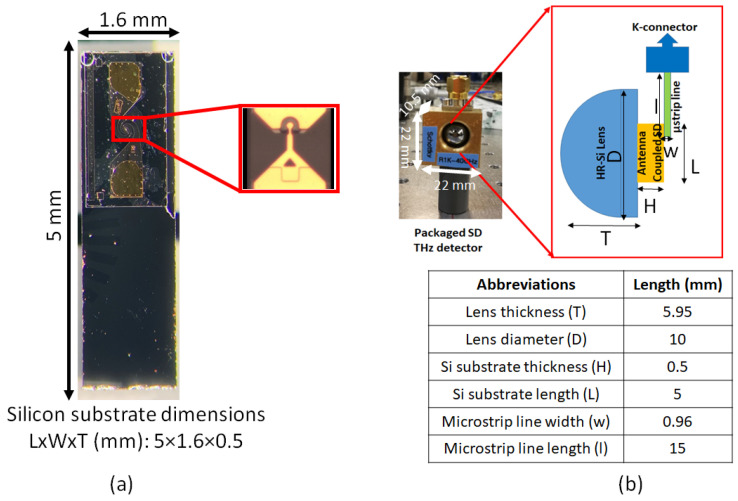
(**a**) Antenna-coupled zero-bias Schottky diode mounted on silicon substrate, inset shows the diode and (**b**) packaged detector in the brass housing. The inset depicts optical and electrical connections of the detectors. Dimensions of the structures are shown in the table.

The antenna-coupled SD is shown in Figure 1a. The diode is mounted on a hyper-hemispherical highly resistive silicon lens with a thickness and diameter of 5.95 mm and 10 mm, respectively. The lens focuses the THz wave at the center of the antenna, where the SD device is mounted, as shown in Figure 1b. The brass housing is used to isolate the device from the unwanted coupling of stray fields and external noise, and protects the device from electrostatic discharge (ESD), which can easily destroy it. The device is electrically connected to the post detection electronics by a broadband microstrip line up to 40 GHz, followed by a K connector at the detector output. The housing of the detector was developed in-house with a special focus on its compactness. This compact, broadband and robust detector operates at room temperature and can easily be used by just plugging it in and playing it to the table-top THz systems as well as at accelerator facilities. Its compact size makes it suitable to be used in a very flexible manner.

## 3. Experimental Setups

In this section, the experimental setups used for the characterization of the SD THz detector are described.

### 3.1. Table-Top Experimental Setup

**Figure 2:**
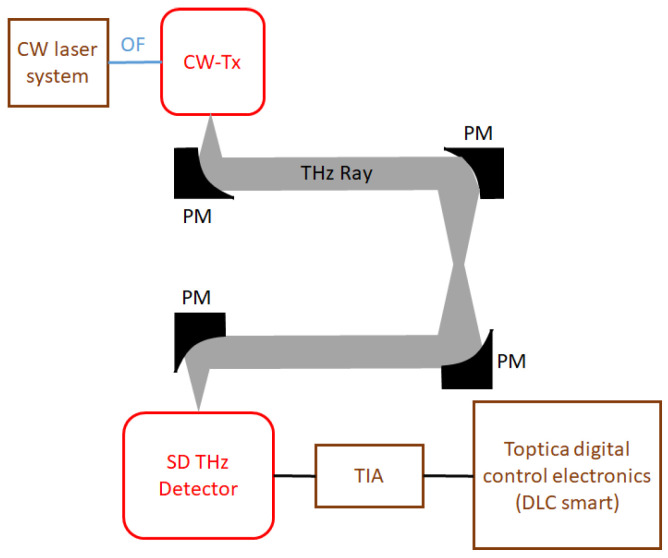
The experimental setup used to characterize the detectors with the table-top CW transmitter from Toptica/HHI. In the setup, OF: Optical fiber, CW-Tx: Continuous wave transmitter, PM: Parabolic mirror, SD: Schottky Diode THz detector and TIA: Trans-impedance amplifier.

The detector was first characterized with a table-top CW system from Toptica (TeraScan 1550). Figure 2 depicts the experimental setup. It is characterized from 0.2 to 1.2 THz using a continuous wave pin-diode-based transmitter as a source. Due to aging, the setup delivers a reduced power of 1.2 and 0.065 μW at 0.5 and 1 THz compared to the specs of the new system (10 μW and 1 μW at 0.5 and 1 THz, respectively). The SD THz detector feeds the rectified signal into a trans-impedance amplifier (PDA-S, TEM Messtechnik) with a gain of $3.3\;\times \;10^{6}$ V/A followed by a lock-in amplifier (Toptica digital control electronics DLC smart) as a post-detection electronic.

### 3.2. FELBE Experimental Setup

**Figure 3:**
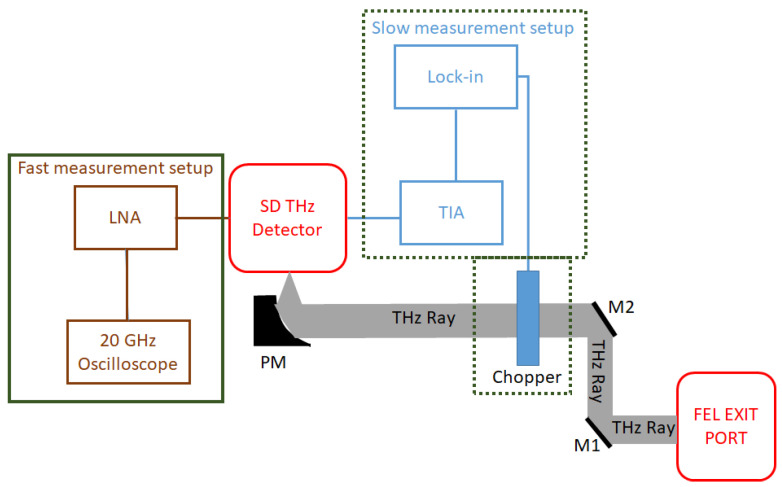
Experimental setup used at FELBE. The solid green box components show the measurement setup for detection of the sub-ns scale signal, while the components in dashed green box shows the slow measurement setup that visualize both slow thermal as well as fast rectification processes. In the setup, PM: Parabolic mirror, LNA: Low noise amplifier, M1 & M2: Mirror and TIA: Trans-impedance amplifier.

In order to characterize the SD detector for its operation limits, and its responsivity and behaviour with respect to high power levels, particularly at several THz, further experiments were carried out at the Free-Electron Laser facility FELBE at HZDR, Dresden, Germany. Figure 3 depicts the experimental setup.

The IV characteristic of Schottky diodes, and thus their rectification responsivity, is known to be strongly temperature (*T*)-dependent. Both the reverse saturation current ($I_{S}\propto T^{2}$) as well as the exponent of the IV characteristics ($I\propto e^{\frac{U-U_{bi}}{nk_{B}T}}$, where *U* is the applied bias and $U_{bi}$ is the Schottky barrier height, *n* is the ideality factor and k${}_{B}$ is the Boltzmann constant) are strongly temperature-dependent. Temperature changes caused by incident THz radiation will thus result in a bolometric detection mechanism.

In order to discriminate temperature-dependent (bolometric) effects from fast rectification processes, two setups were implemented: Using a chopper at a modulation frequency of 113 Hz, the lock in-amplifier-based detection chain (dashed green box in Figure 3) detects all rectifying effects, including those depending on temperature changes caused by the powerful FEL beam. Fast rectification processes on the 20–50 ps scale are recorded with a 20 GHz oscilloscope (Tektronix DPO72004B real-time digital phosphor oscilloscope) (solid green box in Figure 3). As lattice-temperature dependent effects usually require at least nanoseconds (local lattice thermalization that affects the band structure and hence the IV characteristics of the SD) to milliseconds (steady state of heat flow from the hot device to the substrate) to reach equilibrium, slow thermal effects cannot be detected. To increase the detector signal on the oscilloscope, a commercially available low noise amplifier (LNA) from 0.01 to 43.5 GHz with a gain of [37 dB] (mini-circuits ZVA-443HGX+) was used. While the detector is designed to have sufficient electronic bandwidth to detect signal modulations up to 40 GHz, the oscilloscope used for these measurements limited the highest detectable modulation frequency to no more than 20 GHz. Furthermore, the overall noise floor was dominated by the input noise of the oscilloscope. The lock-in measurements at the FEL were performed at 3.082, 4.065, 4.840 and 5.560 THz. Due to technical problems, the fast response measurements with the oscilloscope were performed at 1.99, 3.082 and 4.84 THz only. The average power of the FELBE FEL depends on the wavelength, but can reach several tens of watts at most wavelengths, which is several orders of magnitude higher than the average power of typical table-top THz sources. A set of step attenuators in the beam line are used to regulate the power delivered to the measurement site, as the power of a few watts can easily destroy the SD THz detectors. For calibration purposes, the power was measured with a 3A-P-THz sensor from Ophir Optronics.

One benefit of using the FEL for characterization is the large amount of power and correspondingly high pulse energy (up to 2 μJ) available compared to table-top systems, allowing for testing over a very large range of intensities. This is important for determining the linearity of the response. Another benefit of the FEL is the continuous tunability over a very wide spectral range. Two free-electron lasers (U37 and U100) present at FELBE produce coherent electromagnetic radiation in the range of 5 to 250 μm, covering the frequency range of 1.2 to 60 THz. Both FELs deliver pulses with a repetition rate of 13 MHz. The pulse duration of the FEL scales with the wavelength (i.e., longer wavelengths lead to longer pulses), ranging from less than 1 ps at the shortest wavelength to ∼25 ps at the longest wavelength, while some adjustment of the FEL pulse width is possible [18]. The capability for complete control of the wavelength and power of the FEL beam provides a wide range of parameters for the testing and characterization of the SD THz detectors [26].

## 4. Results and Discussion

The SD THz detector was first characterized in-house with a table-top source and the experimental setup shown in Figure 2. The table-top source used for these measurements from Toptica is continuously tunable from 0.2 to 1.2 THz with an average power decreasing with the THz frequency (e.g., an average power of 47, 1.2 and 0.065 μW at 0.1, 0.5 and 1 THz, respectively).

**Figure 4:**
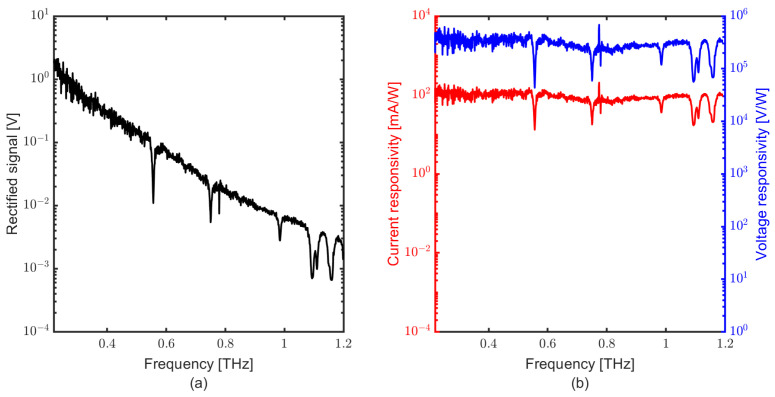
(**a**) Rectified voltage of the SD THz detector with the table-top source, (**b**) current responsivity of the SD on left y-axis (red colour) and voltage responsivity on right y-axis with respect to frequency (blue colour), here including the trans-impedance gain of $3.3\;\times \;10^{6}$ V/A.

Figure 4 shows the results measured with the table-top source. Figure 4a shows the rectified signal rolling off towards higher frequencies primarily due to the roll-off of the pin diode. The responsivity of the detector can be either classified as current responsivity ($R_{I}$) or voltage responsivity ($R_{V})$. In the following sections and graphs, we show the current responsivity of the bare SD detector, while we refer to the voltage responsivity, including the gain of the post detection amplifier.

Figure 4b shows the current responsivity (red graph, left y-axis) and voltage responsivity (blue graph, right y-axis). The features at 0.55, 0.75, 0.98, 1.09, 1.11 and 1.15 THz are water lines. The roll-off observed in the rectified signal (Figure 4a) is always a combination of detector response along with roll-off of the THz source, but almost no roll-off is observed for the detector within the spectral range of this measurement, when calibrated to a Golay cell (from QMC instruments) that was cross-calibrated to a pyroelectric detector (SLT THz20). Still, there is some uncertainty in the order of ±30% on the power. The voltage responsivity of the detector is approximately 500 kV/W with a TIA gain of $3.3\;\times \;10^{6}$ V/A. The current responsivity of 100 mA/W is demonstrated with the table-top system. The calculated responsivity for the table-top system measurements assumes a lock in the peak-to-peak correction factor of ∼3 [37]. This correction factor was experimentally obtained from time-domain acquisitions as the ratio of the full-waveform peak-to-peak response and its fundamental component.

**Figure 5:**
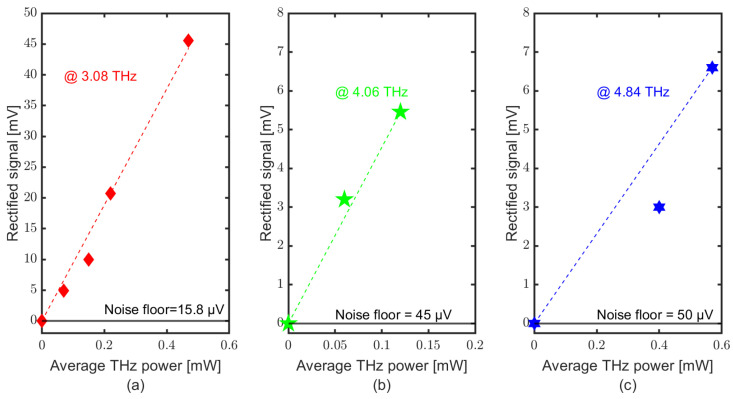
SD THz detector response with the FELBE as source at different frequencies and power using the lock-in measurement technique. (**a**) at 3.08 THz, (**b**) at 4.06 THz and (**c**) at 4.84 THz.

Figure 5 shows the lock-in signal of the detector at different powers and frequencies recorded at FELBE. At 3.08 THz, the measurement is carried out at four power levels: 0.075, 0.15, 0.22 and 0.47 mW, resulting in a rectified signal of 4.9, 10, 20.08 and 45.6 mV, respectively. At 4.06 THz with 0.06 and 0.12 mW of THz power, the rectified signal was 3.2 and 5.46 mV, respectively. At 4.84 THz with 0.4 and 0.57 mW of THz power, the rectified signal was 3 and 6.6 mV, respectively. Due to the limited beam time at the FELBE facility, only a limited amount of data could be taken. The linear trend at 3.08 THz shows that the SD detector did not saturate at the used power levels. We did not attempt to use higher power levels in order to protect the device from destruction.

**Figure 6:**
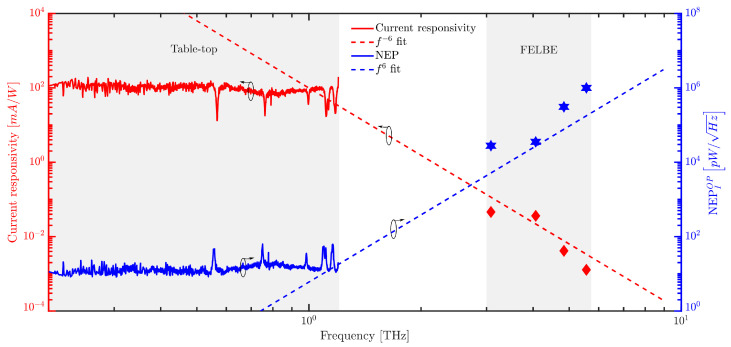
SD THz detector responsivity and NEP comparison with table-top and FELBE using the lock-in measurement technique.

Figure 6, shows the combined current responsivity (in red colour) trend with both table-top source as well as the FEL. The TIA gain of $3.3\;\times \;10^{6}$ V/A with the lock-in amplifier (signal recovery 7270 from AMETEK) measurements was used for both table-top and FEL experiments. At 3.08 THz, a maximum responsivity of 97.02 V/W was observed with a TIA gain of $3.3\;\times \;10^{6}$ V/A, and at 4.065, 4.84 THz and 5.56 THz a responsivity of 45.48, 11.54 V/W and 2 V/W was observed, respectively. We remark that the lock-in amplifier only detects the average signal generated by the SD, while the actual received signal is composed of many, ps-scale short pulses with a THz frequency-dependent pulse length. While a thermal power meter in this case will rather measure the pulse energy deposited within the integration time of the lock-in with little loss, the post detection electronics may partially low pass filter some of the rectified signal, resulting in a reduced signal amplitude. As we do not have any continuous-wave signals available at the FEL wavelengths, we cannot make any conclusive statements at this point and need to leave this aspect for future studies. Further, the interplay of a decreasing antenna radiation resistance of the antenna and the electrodes of the Schottky with increasing frequency and the RC-roll-off leads to an empirical $1/f^{6}$ roll-off of the responsivity which is indicated by the fit in Figure 6. The severe loss of responsivity between the CW and pulsed data may be due to filtering by the post detection electronics, but this phenomenon needs to be enlightened in future work. Figure 6 also shows the NEP in the blue colour on the right y-axis. From 0.2 to 0.6 THz, NEP of 10 pW/$\sqrt{\mathrm{Hz}}$ is obtained. At 1.2 THz, NEP is 17 pW/$\sqrt{\mathrm{Hz}}$. At higher frequencies (FELBE measurements), NEP increases to 0.9 μW/$\sqrt{\mathrm{Hz}}$ at 5.56 THz with the trend of $f^{6}$.

We also took measurements at 7.74 THz with 0.45 mW average power, but the signal was close to the noise floor. We remark that at 7.74 THz, we are fairly close to the Reststrahlen band of GaAs between ∼8 to 10 THz, i.e., there will be a severe attenuation already by its exponential tails. In earlier experiments with the GaA-based HEMT detector, we already observed a strong reduction of the signal around 6.5 THz [17]. This is in line with findings on InP material that feature a fairly similar position of the Reststrahlen band [38].

**Figure 7:**
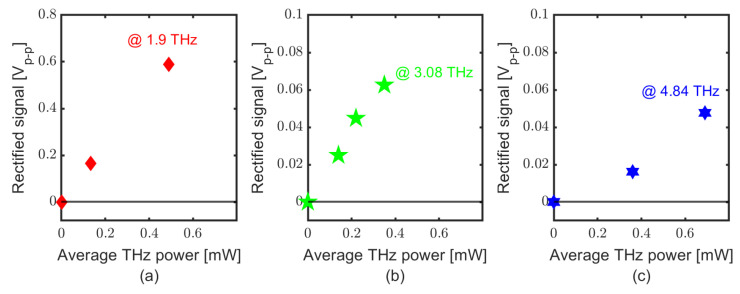
SD THz detector response with FELBE at different frequencies and power using the oscilloscope, (**a**) at 1.9 THz, (**b**) at 3.08 THz and (**c**) at 4.84 THz.

**Figure 8:**
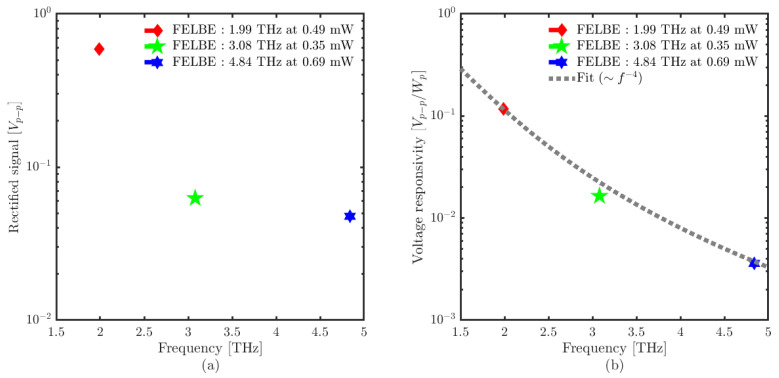
(**a**) Peak–peak rectified voltage; (**b**) voltage responsivity of SD THz detector with FEL using the oscilloscope measurement technique.

Fast rectification processes are recorded with the experimental setup shown in Figure 3, with the post detection electronics (solid green box). Figure 7 shows the peak–peak voltage recorded for several powers at 1.9, 3.08 and 4.84 THz. Similar to the lock-in measurements, no saturation was observed within the measurement accuracy. In order to evaluate the responsivity, the peak power, $P_{p}$, was determined from the average power, $P_{av}$ recorded with the pyroelectric detector as $P_{av}\;=\;P_{p}\cdot \tau_{pls}\cdot \nu_{rep}$, where $\tau_{pls}$ is the (frequency-dependent) pulse duration and $\nu_{rep}\;=\;13$ MHz is the repetition rate of FELBE. Figure 8 shows the peak–peak rectified signal and voltage responsivity of the detector. The maximum obtained voltage responsivity is 0.12 $V_{p-p}$/$W_{p}$ at 1.99 THz, 0.02 $V_{p-p}$/$W_{p}$ at 3.08 THz and 0.0035 $V_{p-p}$/$W_{p}$ at 4.84 THz. The voltage responsivity with the oscilloscope measurement technique shows only a $1/f^{4}$ roll-off, i.e., much less severe than for the lock-in measurements, pointing again to low-pass filter losses of the lock-in intermediate frequency (IF) chain. The responsivity of the detector decreases with the wider spectra (high frequencies) due to the attenuation from the limited IF bandwidth and post detection electronics (20 GHz oscilloscope used for these measurements).

We remark that the peak–peak measurements with the oscilloscope and the lock-in measurements are not easily and directly comparable. First, the oscilloscope post detection electronics contain a power amplifier with considerably low gain of 37 dB whereas the lock-in measurement post detection electronics employ a TIA with $3.3\;\times \;10^{6}$ V/A. Second, the conversion from peak–peak power to average power is frequency-dependent, as the pulse width becomes shorter with the increasing frequency. For the measured pulses, the peak–peak power is about 10,000 times higher than the average power. Third, impedance-matching does not need to be considered for the lock-in measurements, while for the 20 GHz measurements with the oscilloscope, it is a crucial factor. Fourth, the post detection electronics of both alter the read-out voltage. For the oscilloscope traces, none of the pulses are temporally resolved, as the pulses are shorter than the temporal resolution of the oscilloscope. This leads to the broadening of the pulse in conjunction with a peak height reduction. For the lock-in data, the filtering effect of the post detection electronics is unclear.

**Figure 9:**
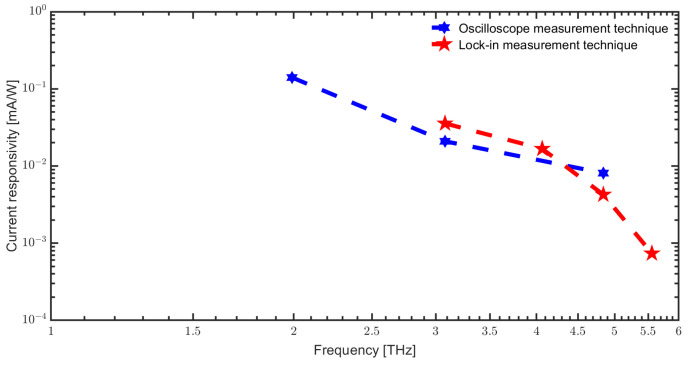
Comparison of current responsivity with oscilloscope and lock-in measurement technique (FELBE measurements).

In [18,36], we have derived the frequency-dependent conversion factor originating from the various imperfections of the setup and compared the estimated average rectified current from the oscilloscope measurements with those from the lock-in. The derived conversion factor is used to compare the two measurement techniques results. Figure 9 shows the comparison between the current responsivity with the oscilloscope and lock-in measurement technique, which shows a very good agreement. We therefore conclude that slow, bolometric thermal detection does not play a noticeable role for the SD detector at least between 3 and 5 THz, where data for both detection mechanisms are available.

In Table 1, we present the comparison of the results obtained in this work with other room temperature operable broadband THz detector technologies at their best operational values. The SD detector characterized in this work demonstrated an optical NEP of 10 pW/$\sqrt{\mathrm{Hz}}$ over the spectral range from 0.2 to 0.6 THz, providing the best performance compared to other similar broadband detection technologies in this frequency range. For the measurements with the table-top system, the optical NEP increases to 17 pW/$\sqrt{\mathrm{Hz}}$ at 1.2 THz, which is still exceptional. Compared to GaAs TeraFET [17], the demonstrated SD THz detector shows, at lower frequencies, a better response (measured with the table-top system); the performance decreases more rapidly at higher frequencies (measured at FELBE). We remark that resonant designs optimized for a specific frequency will outperform broadband detectors. In ref. [39], e.g., the authors have reported a noise floor of 3 pW/$\sqrt{\mathrm{Hz}}$ around 450 GHz. However, these detectors will not perform well over the large frequency range used in this paper.

## 5. Conclusions

In this study, we presented a characterization of a room temperature operable broadband zero-bias Schottky diode THz detector up to 5.56 THz. The characterized SD detector yields an optical noise equivalent power (NEP) of 10 pW/$\sqrt{\mathrm{Hz}}$ from 0.2 to 0.6 THz and 17 pW/$\sqrt{\mathrm{Hz}}$ at 1.2 THz. At 5.56 THz, a NEP of 0.9 μW/$\sqrt{\mathrm{Hz}}$ was demonstrated. For continuous-wave excitation, the voltage responsivity of ∼500 kV/W was constant below 1.2 THz. Measurements at the accelerator facility demonstrated an empirical roll-off of $1/f^{6}$ for the average signal recorded with a lock-in amplifier at the ms-level, while the sub-ns-scale response only rolls-off as $1/f^{4}$. The possible low-pass filtering of the post detection electronics could be the origin of the discrepancy between the roll-offs observed at different time scales. A direct comparison of a THz pulse and a continuous-wave signal at the same frequency would shed light on this aspect.

We remark that the measurements at 5.56 THz and above demonstrated a sharp decrease in responsivity, likely due to the proximity of the Reststrahlen band. However, even at 7.74 THz, the lock-in measurements showed a small signal just above the noise floor at an FEL power of 0.45 mW of power. We expect that the detector will also work above the Reststrahlen band of the detector material. The lock-in measurements (slow measurements) and oscilloscope measurements (fast measurements) demonstrated that thermal effects do not play a significant role at the power levels used in this manuscript. Along with its application at particle accelerators, this detector can be used for other heterodyne measurements, where the radiant THz power from the source is significantly above the detector and post detection electronics’ noise floor.

## Figures and Tables

**Table 1:** Comparison of room temperature broadband THz detectors at their best operational values.

Detector	NEP (pW/$\sqrt{\mathrm{Hz}}$)	Responsivity (V/W)	Operational Frequency (THz)	References
Pyroelectric	2000	56,000	0.14	[40]
Golay cell	140	100,000	-	[41]
SiCMOS TeraFET	20.8	54,000	0.25	[42]
GaAs TeraFET	250	-	0.6	[17]
GaN TeraFET	25.4	-	0.5	[43]
Graphene TeraFET	130	74	0.4	[44]
Schottky barrier diode	11.6	700	0.4–0.6	[45]
Zero-bias Schottky diode	10	500,000	0.2–0.6	This work

## Data Availability

Not applicable.

## Abbreviations

The following abbreviations are used in this manuscript:

CW	Continuous wave
EOS	Electro-optical sampling
ESD	Electro static discharge
HEMT	High electron mobility transistor
HZDR	Helmholtz Zentrum Dresden Rossendorf
FET	Field effect transistor
FEL	Free-Electron Laser
IF	Intermediate frequency
LNA	Low noise amplifier
NEP	Noise equivalent power
OF	Optical fiber
OR	Optical rectification
PM	Parabolic mirror
SD	Schottky diode
TeraFET	Terahertz field effect transistor
THz	Terahertz
TIA	Trans-impedance amplifier
